# How Primary School Children Perceive Tolerance by Technology Supported Instruction in Digital Transformation During Covid 19

**DOI:** 10.3389/fpsyg.2021.752243

**Published:** 2021-09-07

**Authors:** Özge Sakalli, Fahriye Altinay, Mehmet Altinay, Gokmen Dagli

**Affiliations:** ^1^Department of Educational Administration and Supervision, Institute of Graduate Studies, Near East University, Nicosia, Cyprus; ^2^Societal Research and Development Center, Institute of Graduate Studies, Near East University, Nicosia, Cyprus; ^3^Department of Educational Administration and Supervision, University of Kyrenia, Kyrenia, Cyprus; ^4^Educational Administration and Supervision, Near East University, Nicosia, Cyprus

**Keywords:** technology, learning, Covid 19 period, digital transformation, tolerance education

## Abstract

Tolerance education started at an early age in primary schools for a multicultural life. Video-based educational applications provide that child actively participate and learn. This study aims to explore perceptions of primary school children toward tolerance by technology-enhanced learning in Covid 19 period. Online semi-structured interview form was used and the data were analyzed with content analysis. Children developed the universal values such as equality, empathy, not harming themselves, being fair, helping others, happiness, smiling, hugging, respecting, giving gifts, loving people and all living beings without discrimination against disability by technological materials and online education. Future of education relies on tolerance education by incorporating universal values.

## Introduction

The fact that people live in harmony together evokes the concept of tolerance. As a result of the historical processes from the past to the present, the culture, tradition, thought and behavior styles of societies are changing. Living together in a changing environment in a social sense is possible with the education given to individuals. Tolerance training is necessary for the individual to realize his own limits, to live life in line with his own plans, to be aware of the human need to exist in social life and to direct his intelligence toward good (Chistolini, 2017). UNESCO (1994) emphasizing the importance of tolerance education and conducting studies in this field, expresses tolerance education as the development of the new generation's ability to reach independent judgment, critical thinking and ethical reasoning. Sönmez and Aksan (2019) reveals the boundaries, form and quality of relations between people surrounded by values in social life, while tolerance consists of themes related to religion, racial, politics and culture. Hidayat and Nasution (2020) express the definition of tolerance in their studies as a state of respecting and valuing differences, religious beliefs, and differences of opinion, establishing good relations with people, and being able to behave fairly without prejudice. Miller and Sessions (2005) emphasizes that tolerance is a primary value in achieving serenity and peace in societies with diversity such as religious beliefs, culture, race and gender. Lysenko et al. (2020) tolerance is becoming the most up-to-date problematic of the contemporary world, following the globalization process and crises caused by migration. For this reason, in order to develop tolerance awareness in young generations, it is necessary to organize educational programs in schools and to equip those who carry out education with sufficient knowledge and skills.

Nowadays, with these advances, students are digital natives, as they master the technology in this process; teachers, on the other hand, are regarded as digital immigrants as they meet technology later (Bittman et al., 2011). It is stated that students experience real life by watching real life, learning activities are high, and individuals can progress at a low cost according to individual differences) with various course materials used within the scope of distance education such as animations, simulations, sounds, images and videos (Kilit and Güner, 2021).

Due to the Covid-19 epidemic, there is a compulsory transition of schools to distance education throughout the world, and it is stated that the participation in the lessons is quite high as a result of the virtual adaptation of technology in the higher education institution in Bangalore and as a result of the students enjoying these experiences (Shenoy et al., 2020). In their work, Akcil and Bastas (2020) point out the importance of digital citizenship, with the transition from face-to-face education to online education during the Covid-19 pandemic process. In their studies with university students, they concluded that the relationship between digital citizenship behaviors and e-learning attitudes, which are effective in learning willingness in positive way. Unwin (2019) discusses the effects of technology use on future education in his study. It is also stated that the digital content and devices used in education will develop in such a way that each student can take responsibility for and control their own learning, and the increasing need for videos will increase as the content gets richer. McKnight et al. (2016) complies with the principles of student-centered education, in which he assumes an active role in student learning, in collaboration to integrate technology into education, to reach up-to-date learning resources, to have diversity in method, and to enable students to be critical thinking, problem solving and creative at the same time.

In this context, it is thought that the use of video in the course content is important for an effective tolerance education in order to conduct the course in a technology-integrated manner and to ensure permanence in learning. In this direction, the feedbacks from students gain importance. The aim of this research is to report students' experiences of using technology-mediated learning applications for tolerance education. The following questions were examined in this study;

1. How do students define the concept of tolerance?
2. What does use of technology-based learning gain students?
3. What are the gains of students in tolerance education with technology supported education?

## Materials and Methods

This research is a qualitative study conducted to examine the experiences of students and to clarify the use of technology within the scope of tolerance. This research is carried out using phenomenology design. It is a study conducted with phenomenology to reveal the subjective opinions, thoughts and perceptions of the participants about the subject (Schwandt, 2000). The researcher preferred to carry out the applications online with her own classroom due to the fact that she is also a classroom teacher and due to the Covid-19 outbreak. The sample group consists of 17 students studying in class 3 A of the relevant public primary school affiliated to the TRNC MEB in Nicosia in the 2020-2021 academic year. In the study, the student group was chosen in accordance with the “easily accessible case sampling” of the purposeful sampling approach.

The researcher used the interview technique (story telling) or diary, which are among the qualitative data collection methods, to collect the research data. This research consists of two stages. In the first stage, students were presented with a video screening in accordance with the content of tolerance education in the course, which was taught online using Zoom in web-based distance education, and in order to reveal student perceptions on this subject, both focus group meetings were held during the course and online interviews were made with students using a pre-formed semi-structured interview form after the course. Qualitative data were analyzed through thematic analysis. The coding system was used to classify themes. Since the principle of confidentiality was taken into account in the research, while the participants' opinions were included in the findings section, the students were presented in the form of codes S-1, S-2, …

## Results

### Finding 1: Students' Views on the Concept of Tolerance

Table 1 presents the findings that emerged in line with the data obtained as a result of the interviews with the students. 17 students participated in the study. Participant findings are as follows: Empathy (f: 2), Equality (f: 1), Justice (f: 1), Helpfulness (f: 6), Understanding (f: 2), Respect (f: 8), Affection (f: 6), Being Indulgent (f: 7), Behaving well (f: 14), Respecting differences (f: 2), Generous (f: 1), Nice talk (f: 2), Discreet (f: 1), Honest (f: 1), Calm (f: 3), Give value (f: 2), Happiness (f: 4), Smiling (f: 2). Students put forward 65 different views to express the concept of tolerance.

**Table 1:** Students' views on the concept of tolerance.

**Tolerance concept**	***F***	**%**
Empathy	2	3%
Equality	1	2%
Justice	1	2%
Helpfulness	6	9%
Understanding	2	3%
Respect	8	11%
Affection	6	9%
Being tolerant	7	11%
Behaving well	14	21%
Respecting differences	2	3%
Generous	1	2%
Nice talk	2	3%
Discreet	1	2%
Honest	1	2%
Calm	3	5%
Give value	2	3%
Happiness	4	6%
Smiling	2	3%
Total	65	100%

Most of the students explain the concept of tolerance as “Behaving well.” Q-3 “*When we treat our friends well without offending them, they are also happy and they mutually treat us well.”* Q-11: “*We should get everyone to play while we are playing at school. For example, if we do not let our friends play, they may cry and get upset. Also, mocking is a very bad thing. I treat my friends well, being nice is a very nice thing.”* expressions about behaving well are used.

Another finding is that tolerance is expressed as “being tolerant.” Q-14: “*If my friend asks me a lesson that he does not understand, I will tolerate him and help him in his lesson. I speak without yelling at him.”*

Regarding the finding on “Respect,” Q-2: “*Not all people are the same. Some are music lovers, some are football lovers, people do not necessarily have to do the same hobby. I have to show respect,”* statement is included.

### Finding 2: Opinions on the Benefit Use of Technology-Based Learning for Students

The findings obtained from the perceptions of the students about the benefit of using technology-based learning are presented in Table 2. A total of 29 opinions regarding participant findings are presented as; Positive effect on technology use skill (f: 7), Easier understanding of the subjects (f: 5), Lessons becoming interesting (f: 8), Having a fun class environment (f: 6), Being motivating for learning (f: 2), Taking an active role (f: 1).

**Table 2:** Student views on the benefit use of technology-based learning for students.

**Contribution of technology-based learning**	***F***	**%**
Positive effect on technology use skill	7	24%
Easier understanding of subjects	5	17%
Lessons becoming interesting	8	28%
Having a fun class environment	6	21%
Being motivating for learning	2	7%
Taking an active role	1	3%
Total	29	100%

From the opinions, it is seen that “lessons become interesting” is the majority. Q-7 “*It was great that the lesson was handled with video. I watched curiously what would happen. I wish all the lessons were taught with video.”*

Among the findings, “the positive effect of technology use skills” is one of the opinions agreed. Q-8: “*We used to teach our lessons in the classroom before, but if it is, we do lessons on the tablet. We use technological things we haven't used before, we learn new things. I used to only use my tablet to play games.”*

### Finding 3: Technology-Supported Education and Students' Views on the Achievements in Tolerance Education

In Table 3, the opinions of the participants regarding the use of technology-supported education in tolerance education are presented in the form of themes. Findings of participant; Positive contribution to academic development with regard to tolerance (f: 11), Enables students to interact with regard to tolerance (f: 3), Raising awareness on tolerance (f: 6), Preparing students for social life with regard to tolerance (f: 2) and 22 topics are presented. The most common statement is about the theme “positive contribution to academic development with regard to tolerance.” Q-5: “*The video I watched impressed me… I realized how important it is to be tolerant and understanding, respecting the disabled and everyone.”*

**Table 3:** Students' views on technology-supported teaching and achievements in tolerance education.

**Technology-supported education and achievements in tolerance education**	***F***	**%**
Positive contribution to academic development with regard to tolerance	11	50%
Enables students to interact with regard to tolerance	3	14%
Raising awareness on tolerance	6	27%
Preparing students for social life with regard to tolerance	2	9%
Total	22	100%

Regarding the theme of “raising awareness on tolerance,” Q-12: “*I did not know what tolerance was before. We have to show respect to my friend, whether he/she is successful or not at mathematics, and everyone who is dark or light in skin color.”*

## Discussion

In this study, when the findings obtained on how technology-enhanced learning improves students' skills in tolerance education, students defined tolerance as follows: Empathy, Equality, Justice, Helpfulness, Understanding, Respect, Affection, Being Indulgent, Behaving well, Respecting differences, Generous, Nice talk, Discreet, Honest, Calm, Give value, Happiness, Smiling. It is seen in the study of Kaygısız (2019) that similar findings were obtained in the form of loving, being respectful, sharing and doing good. In another study, Aslan (2019) reveals the perceptions of primary school 4th grade students within the framework of tolerance and respect, and the findings are similar to helping and sharing based on love and respect. In Aslan and Aybek (2020) studies, it is similar to this study in terms of students' perception about tolerance which suggests that in order to be tolerant, it is necessary to be helpful, respectful and not to break anyone's heart. Ersoy (2016) explains that students perceive tolerance as helping, loving and behaving well. Wainryb et al. (2004) found in their study on children in the first childhood period that the level of tolerance was low as the age got smaller. Tillman (2000) shows that increasing violence and social problems in children stem from lack of respect and tolerance, and studies show that the most effective way for parents and educators to cope and overcome with these problems is to teach values. Lazovsky (2007) concludes that the participants' communication skills such as showing respect for their thoughts and expressing their feelings to a person who is not of the same opinion can improve.

The findings obtained from students' perceptions about the benefits of using technology-based learning are presented under themes such as the positive effect of the technology use skill, the easier understanding of the subjects, the engaging lessons, an entertaining lesson environment, being motivating for learning, and taking an active role. With the use of technology in tolerance education and the presentation of the lecture in which the audibility and visuality in the videos are added, the attention and interest of the students becomes more intense during the lesson and it is seen that the learning is more permanent. Kenar (2012) indicated that pictures, sound, animation and video in multimedia presented in teaching are beneficial in permanent learning, as they affect many senses. In another study, Levy and Yupangco (2008) stated that the materials used in teaching have a positive effect on learning since they are effective on visual perception. In the study of Demuyakor (2020), it is found that students in higher education institutions perceive the course content for online learning as effective and useful during the Covid-19 process, and they are also satisfied with the use of materials such as texts, software and videos recommended by the teacher. Özmen (2004) stated in his study that the use of technological tools in the lesson increases the interest of individuals in the lesson, motivates them and creates permanent behavioral changes, and that it will be beneficial to get help from technology in the lesson to bring back the old information in the memory. In Taşlibeyaz (2019) study, it is concluded that video blogs attract students' attention to the lesson and facilitate learning.

In this study, students' opinions revealed by using technology-supported teaching in tolerance education under main topics as follows; positive contribution to academic development on tolerance, enabling students' interaction with regard to tolerance, raising awareness about tolerance, and preparing students for social life with regard to tolerance. Eryilmaz and Salman (2014) stated that according to student perceptions, content such as videos, animations and e-books used in e-content facilitates learning and increases success. In Coşkunserçe (2020) research, it is pointed out that the lesson becomes interesting and fun and the participants develop a positive attitude toward values education by using video blogs in values education. It was also stated that these positive attitudes indicate that the course is successful. Basar and Cangal (2021) states that, with the use of new technologies in teaching, the teaching process will provide students with permanent learning and student success will increase.

In this study, students' views on tolerance provide a universal insight with their expressions such as equality, empathy, not harming themselves and world, being fair, helping others, happiness, smiling, hugging, respecting, giving gifts, loving people and all living beings without discrimination. In addition, it is concluded that the participants can take on a more active role while learning thanks to the technological materials used in the lesson, that the use of technology improves their skills, they grasp the subjects more easily, they take an attractive and active role in the lesson, and they are motivated to understand the concept of tolerance with a cooperative and entertaining lesson environment.

Education systems around the world are beginning to be insufficient to address the situations that arise in the face of globalization and technological innovations. Increasing population mobility, increase in the number of immigrants and refugees create diversity in students. Social exclusion and marginalizing behaviors among students that emerge in this situation constitute a problem of tolerance. In order to overcome these problems, education can help students prepare for social life and gain competence in cultures (Isac et al., 2018). Tolerance awareness can be gained through education at schools. In schools, four basic ways are suggested for students to be informed about tolerance and to develop attitudes. These four ways as explained as cognitive complexity, communication with others, socialization of values and identity formation (Janmaat et al., 2018). Aydin and Gürler (2014) state that it is not enough for individuals to only achieve academic success today, but also that it is necessary to acquire behaviors and skills that are important in social life. They that these behaviors and attitudes that they cover many issues as being honest, showing respect and love, sharing, establishing healthy communication, being attentive in their relationships, adopting the rules of courtesy, not being biased, being able to cooperate, being disciplined at work, and avoiding violence.

Bilgin et al. (2018) stated that the transfer of tolerance in families and schools, which form the basis of society, is realized through communication. According to the data obtained, they concluded that communication within the family affects tolerance and is effective in the democratic development of the school and society. Therefore, in order to create tolerance awareness with the participation of families, benefits can be gained by integrating technology and organizing online seminars.

## Data Availability Statement

The original contributions presented in the study are included in the article/supplementary files, further inquiries can be directed to the corresponding author/s.

## Ethics Statement

The studies involving human participants were reviewed and approved by Near East University Ethical Committee. The patients/participants provided their written informed consent to participate in this study.

## Author Contributions

ÖS conceived of the study, participated in the design, data collection, analysis for the study, and drafted the manuscript. FA, MA, and GD participated in its design, data collection, and contributed to drafts of the manuscript. All authors read and approved the final manuscript.

## Conflict of Interest

The authors declare that the research was conducted in the absence of any commercial or financial relationships that could be construed as a potential conflict of interest.

## Publisher's Note

All claims expressed in this article are solely those of the authors and do not necessarily represent those of their affiliated organizations, or those of the publisher, the editors and the reviewers. Any product that may be evaluated in this article, or claim that may be made by its manufacturer, is not guaranteed or endorsed by the publisher.
